# MicroRNAs and their signaling pathway in mycosis fungoides

**DOI:** 10.1097/MD.0000000000029248

**Published:** 2022-06-24

**Authors:** Zhiyuan Sun, Xiaona Yao, Xing Ding, Xun Li, Xuewen Tian

**Affiliations:** Shandong Sport University, Jinan, Shandong Province, China.

**Keywords:** de-expression, mircoRNA, mycosis fungoides, signaling pathway, STAT

## Abstract

**Background:**

Oncogenic microRNAs, a kind of stable epigenetic inhibitors, often deregulated in Mycosis fungoides (MF) which affect the skin and tend to transform and spread.

**Results:**

Previous studies investigating the de-expression of microRNA in MF patients skin biopsies identified that they were not only regulated by signaling pathway, but also regulated other signaling pathway. Furthermore, studies have elucidated the molecular mechanisms of the STAT signaling pathway that can promote a great diversity of miRNA expression via cytokine binding receptors, activating Janus kinase-3 and STAT proteins. But some non-STAT signaling pathway with mircoRNA de-expression in MF was incomplete.

**Conclusion:**

Taken together, these studies demonstrate that microRNA may be used as the prognosis, progression and diagnose of MF, as they can not only control MF cell proliferation, but also induce MF cell apoptosis.

## 1 Introduction

Advanced mycosis fungoides (MF) or Sézary syndrome, major variants of cutaneous T-cell lymphoma (CTCL), are associated with–40% to 47% 5-year survival.^[1]^ The annual incidence of primary cutaneous lymphomas is estimated to be 1:100,000, of which CTCL accounts for approximately 75% of cases; therefore, CTCL is a common type of primary cutaneous lymphomas.^[2,3]^ Through the clonal proliferation of skin-invasive mature T-lymphocytes, CTCLs are characterized as non-Hodgkin's lymphomas.^[2]^ Mycosis fungoides (MF) is a common and indolent form of CTCL and is characterized by patches, plaques, or tumors containing epidermotrophic malignant CD4+CD45 RO+ helper/memory T-cells.^[4]^ In the primary stage, MF appears as a flat erythromatous skin lesion and resembles non-malignant psoriasis or eczema and can last for several years.^[4]^ In later stages, tumor cells spread to other parts of the body as a fatal outcome. MF can develop into a leukemic variant, Sézary syndrome, in which cancer T-cells appear in the skin and blood, or shift to large cell lymphoma.^[5]^ Skin cytokines orchestrate inflammation through their impact on the expression and function of other cytokines, and their downstream effectors (such as STAT and SOCS proteins, microRNA) are frequently observed.^[6,7]^ MicroRNAs (miRNAs) are important molecular markers of MF progression and diagnose.^[8]^

miRNAs are a class of small non-coding RNAs with length of 18 to 22nt that are ubiquitous in eukaryotes and can regulate protein expression at the mRNA level.^[9–11]^ Many studies have reported that MF alters miRNA expression, including reduced miR-191, miR-223, and miR-342, and increased miR-155.^[12]^ These microRNA expression changes influence or influence signaling pathways such as STAT3, STAT5, or p53/Akt et al.^[13,14]^

## 2 MicroRNA expression and MF

It is important to understand how microRNA expression changes influence MF cell proliferation in order to develop them as a new target gene for the prevention and treatment of cancer. From Table 1, the expression of about 12 miRNAs whose function and characteristics were further analyzed by reliable experiments up-or down-regulated in MF skin biopsies or cells. Six miRNA expression upregulates and belongs to an oncogenic molecule. Another 6 miRNAs are downregulated and belong to the suppressor gene. Eight miRNAs can be used to evaluate MF progression; miRNA-122 and miRNA-214 are molecular markers of MF prognosis.^[14,15]^ Moreover, miR-155, miR-203, and miR-205 in patient peripheral blood can be used as diagnostic markers,^[16,17]^ that discriminated between malignant and benign skin inflammation with an accuracy of more than 90%.^[18]^

**Table 1:** Recent microRNA and mycosis fungoides Studies.

MiRNA type	Year	Biomarker use	Sample	Target/Pathway	Expression	Function	Ref.
miR-93	2021	Progression	Malignant T cells lines	p21	down	Oncogenic molecule	^[40]^
miR-195–5p	2020	Progression	Skin biopsies and cell lines	ARL2	down	Suppressor gene	^[41]^
miR-106b	2020	Prognosis	Skin biopsies	P21/TXNIP	up	Oncogenic function	^[42]^
miR-337	2019	/	Malignant and non-malignant T cell lines	JAK/STAT	up	/	^[36]^
miR-214	2019	Diagnose	CD4^+^ T cells	TWIST1/BRD4/miR-214	up	Oncogenic molecule	^[31]^
miR-155	2018	Diagnose	Malignant T-cell lines (MyLa2059, MyLa3675 and MyLa2000) and HH cells	SATB1/GATA3	up	Oncogenic molecule	^[25]^
miR-155	2018	Diagnose	HUT102, HUT78 and HH cell lines	JAK/STAT, MAPK/ERK and PI3K/AKT	up	Oncogenic molecule	^[16]^
miR-155	2017	Diagnose	Peripheral blood	/	up	oncogenic Molecule	^[16]^
miR-203, R-205	2017	Diagnose	Peripheral blood	/	down	Suppressor gene	^[43]^
miR-150	2017	Progression	Skin biopsy, HH, HUT78, and MJ cell lines	CCR6	down	Suppressor gene	^[43]^
miR-214	2017	Prognosis	Skin biopsies	FCRL-3, Tox	up	/	^[15]^
miR-21	2016	Progression	Skin biopsies	STAT5/JAK3	up	Oncogenic Molecule	^[27]^
miR-34a	2016	Progression	Skin biopsies	p53	up	oncogenic Molecule	^[32]^
miR-29a	2016	Progression	Skin biopsies	p53	down	Suppressor gene	^[32]^
miR-17–92 cluster	2016	Progression	Skin biopsies	/	up	Oncogenic function	^[44]^
miR-16	2016	Progression	Skin, My-La, MJ, HUT102, HH, and HUT78	miR-16/p21/Bmi1	down	Suppressor gene	^[35]^
miR-22	2015	/	MyLa2059, MyLa2000, PB2B, MyLa1850 and MySi cell lines	Jak3/STAT3/STAT5	down	Suppressor gene	^[13]^
miR-223	2014	Progression	Skin biopsies, HH and Hut-78 cell lines	TOX	down	Suppressor gene	^[30]^
miR-155	2014	Progression	Skin biopsies	STAT4	up	Oncogenic molecule	^[21]^
miR-155	2013	/	Skin biopsies	STAT5/BIC	up	Oncogenic molecule	^[20]^
miR-122	2012	Prognosis	Skin, MyLa2000, SeAx and Hut-78 cell lines	p53/Akt	up	Oncogenic molecule	^[14]^
miR-21	2011	Progression	SeAx cell line	STAT3	up	Oncogenic molecule	^[28]^

## 3 MicroRNA and STAT signaling pathway

**Figure 1:**
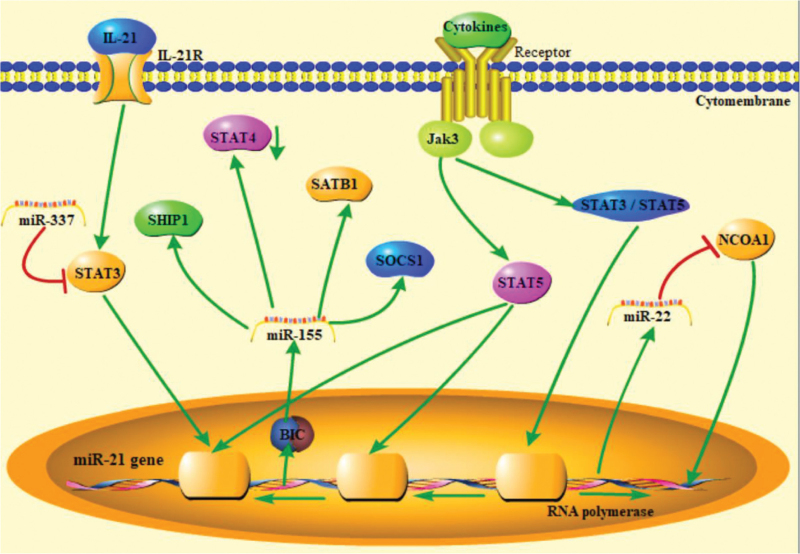
MicroRNA and STAT signaling pathway.

The expression and function of STAT3, STAT4, and STAT5 have been extensively studied in MF, and these genes appear to play an important role in disease pathogenesis and can be used as important prognostic markers. The effectors (STAT3 and STAT5 et al) and the up-stream Interleukin-2 receptor common gamma chain, the associated Janus kinase-3 (Jak3) have attracted substantial interest. Interleukin-2 receptor common gamma chain-signaling cytokines, including IL-2, IL-4, IL-7, IL-15, and IL-21 are implicated in early pathogenesis and constitutive.^[13,19]^ As shown in Figure 1, the deregulation of Jak3/STAT3/STAT5 signaling in MF cells can repress the expression of the gene encoding miR-22, which targets the transcriptional co-activator NCoA1.^[13]^

The expression of miR-155 is enhanced in the affected skin of MF patients compared to that in healthy individuals.^[8,18]^ The host gene of miR-155 is BIC, which is transcribed by STAT5.^[20]^ Upregulated miR-155 binds and cleaves its target gene, STAT4, in advanced MF patients skin biopsies.^[21]^ The PI3K/AKT, JAK/STAT, and MAPK signaling pathways negatively regulate signaling through miR-155, directing inositol phosphatase SHIP1 and SOCS1.^[22–24]^ When JAK3 phosphorylates STAT5, it translocates to the nucleus and initiates the transcription of miR-155. Upregulation of miR-155 leads to inhibition of SATB1 and inhibition of IL-5 and IL-9 expression.^[25]^ Oncogenic miR-155 appears to contribute to the cancerous phenotype of MyLa and MJ cells by interrupting the activation of the G2/M checkpoint.^[26]^ STAT5 is not only a driver of miR-155 but also regulates miR-21 expression, which has been linked to disease progression interconnecting the JAK/STAT pathways as a key regulator of miRNAs in CTCL.^[27]^ STAT3 has been regarded as a promising therapeutic target for many years in Se’zary syndrome and can increase miR-21 expression by IL-21 activating IL-21R and STAT3, and then miR-21 inhibits Se’zary syndrome cell apoptosis.^[28]^

## 4 MicroRNA and other signaling pathway

**Figure 2:**
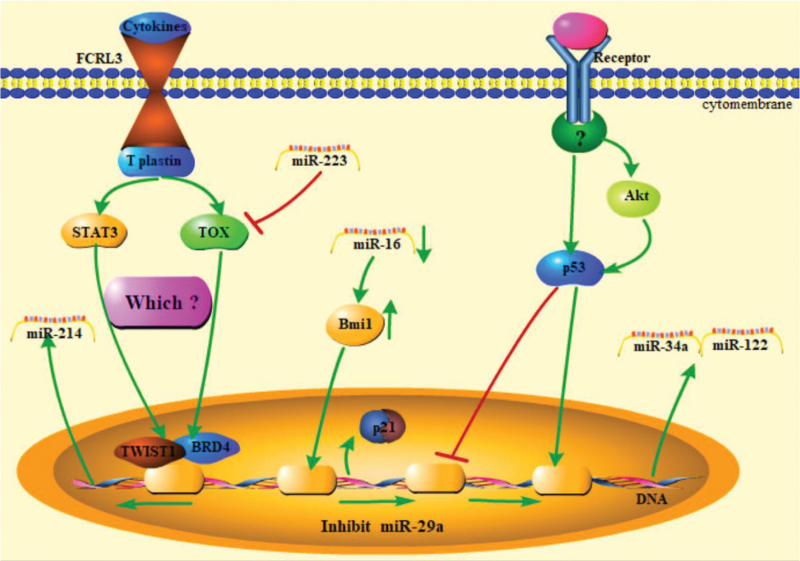
MicroRNA and signaling pathway participated by p53, STAT3, and TOX.

The deregulation of signaling pathways, including STAT, Src kinases, c-Myc, COX-2, NFκB, GATA3, TOX, and embryonic stem cell regulators, appears to play an important role in pathogenesis.^[13,29]^ As shown in Figure 2, Fc receptor-like protein 3, along with T plastin, GATA-3, Tox, and miR-214, was significantly higher in Se’zary syndrome patients.^[15]^ Based on the above results, we hypothesized that GATA-3 or/and Tox were activated by activating Fc receptor-like protein 3 and T plastin, and then they or/and enhanced miR-214 transcription. The protein level of Tox is regulated by miR-223.^[30]^ Kohnken et al showed that miR-214 levels are significantly higher in purified CD4þ neoplastic T cells from patients with MF than from healthy donors. Further studies have proposed that the TWIST1 and BRD4 complex regulate miR-214 expression.^[31]^

Deregulation of the p53 signaling pathway was also found in MF patient skin biopsies or cells, including My-La, MJ, HUT102, HH, and HUT78.^[32]^ The up-regulated miR-122 inhibits tumor cell apoptosis by Akt/p53 signaling pathway,^[14]^ similarly miR-34a expression level was also increased by p53 signaling pathway in Se’zary syndrome patients skin biosies.^[32]^ However, miR-29a expression was lower in MF patients than in healthy patients because of p53 signaling pathway inhibition.^[32]^

Earlier studies did not detect changes in p16 expression in all MF cells until 2016.^[33,34]^ Kitadate et al speculated that miR-16 directly or indirectly suppresses Bmi1, thereby enhancing p21 expression in MF cells based on their experiments results.^[35]^ It has been confirmed that microRNAs can directly regulate receptor proteins. The miR-150 in MF cells was upregulated and combined with the C-C chemokine receptor 6 “seed sequence” mRNA of the 3-untranslated region (3-UTR) in advanced MF.

STAT3 expression can be significantly downregulated following transfection with the miR-337 mimic, which potentially targets the 3-UTR of STAT3.^[36]^ MF progression can be estimated by analyzing the upregulated expression of miR-155, miR-146a, 146b-5p, miR-342–3p, and let-7i∗ and downregulated expression of miR-203 and miR-205.^[37]^ Subsequent studies confirmed that miRNAs are potentially valuable tools for the evaluation of disease progression in MF.^[38,39]^

## 5 Conclusions and perspectives

Collectively, the findings discussed in the present review provide novel insights into the effect of microRNAs on MF cells, which supports new concepts for the prognosis, progression, and diagnosis of MF. As shown in Table 1, some miRNAs were upregulated in the skin biosies of MF patients; however, some miRNAs were downregulated. These miRNAs can be used as biomarkers for disease diagnosis and as therapeutic targets.

MF cell proliferation and apoptosis involve the functional cooperation of many signaling molecules. As summarized in Figure 1, STAT signaling pathways, including STAT3, STAT4, and STAT5, can promote a great diversity of miRNA expression via cytokine binding receptors, activating Jak3 and STAT proteins. Transcripted microRNAs regulate MF cell proliferation and apoptosis via other signaling pathways or target molecules.

MicroRNA transcription can also be regulated by another signaling pathway, as shown in Figure 2. However, these signaling pathways can regulate microRNA expression and can be regulated by microRNAs. It is not clear at the moment that some important molecules are involved in this pathway. STAT3, p53, TOX, and Bmi1 participate in these pathways and play an important role.

These results indicate that cocktail therapy with microRNA and other drugs may greatly reduce the risk of MF cell metastasis in all types. The significance of miRNA as a molecular marker may be used for prognosis, progression, and diagnosis of MF in the future. Further studies are required to determine which of the signaling pathways or miRNAs is most important for the treatment or diagnosis of MF. In addition, many previous studies have not established an integrated non-STAT signaling pathway. Future studies investigating whole signal regulation in MF may provide further insight into the mechanisms underlying its activity.

## Author contributions

**Conceptualization:** Xiaona Yao, zhiyuan Sun.

**Data curation:** Xiaona Yao, zhiyuan Sun.

**Formal analysis:** Xiaona Yao, zhiyuan Sun.

**Funding acquisition:** Xiaona Yao, Xuewen Tian, Xun Li.

**Resources:** Xing Ding.

**Writing – review & editing:** Xiaona Yao, zhiyuan Sun.
